# Supplementary material for: Content-rich biological network constructed by mining PubMed abstracts
Source: BMC Bioinformatics. 2004 Oct 8;5:147. doi: 10.1186/1471-2105-5-147 (PMC528731; doi:10.1186/1471-2105-5-147)
Supplement: Additional File 2 — The original results of the above study (non-essential files are deleted to keep the file size under the limit set by BMC bioinformatics). [file 1471-2105-5-147-S2.bz2 › chilibotAdditionalFile2/dip30/375ID3037029E9781/html/NGFR_NGFB.html]

 


 **NGFR** and **NGFB** 
  
Found 1676 abstracts in PubMed, retrieved 30.  
 

 What does Google say? 
 PDF only 
| .edu only 

---

**Interactive relationship** (e.g. stimulation, inhibition, etc)

**Inhibitory relationship**- Disruption of the phosphoinositide 3 kinase PI 3 kinase pathway, one of the signaling pathways downstream from NGF  [ **NGFB** ]  receptor  [ **NGFR** ]  binding, completely eliminates NGF  [ **NGFB** ]  effects on mitochondrial behavior in axons.  Ref: 12756280 J Exp Biol, 2003
- Pathways responsible for transducing the sensitisation of TRPV1 by TrkA, the NGF  [ **NGFB** ]  receptor  [ **NGFR** ] , were characterised by observing the effects of inhibitors of key members of NGF  [ **NGFB** ]  activated second messenger signalling cascades.  Ref: 12815188 J Physiol< MedlineTA>J Physiol, 2003
**Neutral relationship**- NADE p75NTR  [ **NGFR** ]  associated cell death executor is a p75NTR  [ **NGFR** ]  associated protein that mediates apoptosis in response to NGF  [ **NGFB** ]  by interacting with the cell death domain of p75NTR  [ **NGFR** ] .  Ref: 12852261 Vitam Horm, 2003
- Here we show that survival of sympathetic neurons supported by NGF  [ **NGFB** ]  on distal axons requires the kinase activity of the NGF  [ **NGFB** ]  receptor  [ **NGFR** ] , TrkA, in both distal axons and cell bodies.  Ref: 12848932 Neuron, 2003
- The p75 neurotrophin receptor p75NTR  [ **NGFR** ]  is a member of the tumor necrosis factor receptor TNFR superfamily, and can mediate both cell survival and cell death in response to nerve growth factor  [ **NGFB** ]  NGF  [ **NGFB** ] .  Ref: 12852261 Vitam Horm, 2003

**Non-interactive relationship** (e.g. studied together, co-existance, homology, etc.)

- Human nerve growth factor  [ **NGFB** ]  receptor  [ **NGFR** ]  and cytosine deaminase fusion genes.  Ref: 12869218 Hum Gene Ther, 2003
- SHB mediates certain responses in platelet derived growth factor PDGF receptor, fibroblast growth factor FGF receptor, neural growth factor NGF  [ **NGFB** ]  receptor  [ **NGFR** ]  TRKA, T cell receptor, interleukin 2 IL 2 receptor and focal adhesion kinase FAK signaling.  Ref: 12776987 Curr Mol Med, 2003
- Expression and Activation of the Nerve Growth Factor  [ **NGFB** ]  Receptor  [ **NGFR** ]  TrkA in Serous Ovarian Carcinoma.  Ref: 12796393 Clin Cancer Res< MedlineTA>Clin Cancer Res, 2003
- CONCLUSIONS The results indicate that coexpression of both low and high affinity NGF  [ **NGFB** ]  receptors are not only more efficient in restoration of NGF  [ **NGFB** ]  induced differentiation pathway, but also be able to activate the pro apoptotic activity of low affinity NGF  [ **NGFB** ]  receptor  [ **NGFR** ]  and make the tumor cells become NGF  [ **NGFB** ]  dependent and irreversibly differentiated.  Ref: 12877805 Chin Med J (Engl), 2003
- We propose that DRG central processes arborize extensively in both NGF  [ **NGFB** ]  transgenic and nontransgenic p75NTR  [ **NGFR** ]  hypomorphic mice in order to compensate for the loss of DRG neurons and restore dorsal horn innervation to normal levels.  Ref: 12887413 Eur J Neurosci, 2003
- A murine model of X linked chronic granulomatous disease X CGD, an inherited immune deficiency with absent phagocyte NADPH oxidase activity caused by defects in the gp91 phox gene, was used to evaluate a bicistronic retroviral vector in which expression of human gp91 phox and a linked gene for DeltaLNGFR, a truncated form of human low affinity nerve growth factor  [ **NGFB** ]  receptor  [ **NGFR** ] , are under the control of a spleen focus forming virus long terminal repeat LTR.  Ref: 12804147 Hum Gene Ther, 2003
- In addition, tumors were examined for expression of vimentin, cytokeratin, smooth muscle actin, desmin, melan A, neural cell adhesion molecule, S 100, glial fibrillary acidic protein, nerve growth factor  [ **NGFB** ]  receptor  [ **NGFR** ] , and collagen type IV.  Ref: 12824506 Vet Pathol, 2003
- The 75 kDa neurotrophin receptor p75NTR  [ **NGFR** ]  and two neurotrophin receptor homologs NRH1, NRH2 constitute a subfamily of the nerve growth factor  [ **NGFB** ]  tumor necrosis factor receptor superfamily.  Ref: 12843241 J Neurosci, 2003
- However, NGF  [ **NGFB** ]  rescue from growth factor withdrawal requires NGF  [ **NGFB** ]  signalling through the high affinity tyrosine kinase receptor TrkA, while NGF  [ **NGFB** ]  rescue from TNFalpha promoted cell death requires NGF  [ **NGFB** ]  signalling through the low affinity p75NTR  [ **NGFR** ]  receptor.  Ref: 12882321 Aging Cell, 2003
- In this review, we focus on the proapoptotic actions of NADE that regulate p75NTR  [ **NGFR** ]  signaling in response to NGF  [ **NGFB** ] .  Ref: 12852261 Vitam Horm, 2003
- Expression and Role of Low Affinity Nerve Growth Factor  [ **NGFB** ]  Receptor  [ **NGFR** ]  p75 in Spinal Motor Neurons of Aged Rats following Axonal Injury.  Ref: 12876432 Dev Neurosci, 2003
- Two genetically modified mouse strains were used transgenic mice that overexpress NGF  [ **NGFB** ]  in the CNS under the control of the glial fibrillary acidic protein promoter, and p75NTR  [ **NGFR** ]  exon III null mutant mice that express a hypomorphic form of this receptor.  Ref: 12887413 Eur J Neurosci, 2003
- In this paper, genetic constructs were designed to express the CD enzyme fused to the transmembrane and extracellular domains of the human nerve growth factor  [ **NGFB** ]  receptor  [ **NGFR** ]   **NGFR** , thus allowing for positive identification of transduced cells by flow cytometry and positive selection by magnetic bead technology.  Ref: 12869218 Hum Gene Ther, 2003
- the lack of NGF  [ **NGFB** ]  receptor  [ **NGFR** ]  expression on some astrocytes suggests heterogeneity of astroglia population.  Ref: 12849738 Neuroscience, 2003
- High affinity NGF  [ **NGFB** ]  receptor  [ **NGFR** ]  in the rat spinal cord during acute and chronic phases of experimental autoimmune encephalomyelitis a possible functional significance.  Ref: 12825322 Arch Ital Biol, 2003
- After 2 weeks of treatment of PLA cells with isobutylmethylxanthine, indomethacin, and insulin, about 20 to 25 percent of the cells differentiated into cells with typical neural morphologic characteristics, accompanied by increased expression of NSE, vimentin, and the nerve growth factor  [ **NGFB** ]  receptor  [ **NGFR** ]  trk A.  Ref: 12711954 Plast Reconstr Surg, 2003
- Thus, a chimeric receptor was created that possessed the extracellular and transmembrane domains of the nerve growth factor  [ **NGFB** ]  NGF  [ **NGFB** ]  receptor  [ **NGFR** ]  TrkA and the cytoplasmic domain of PDGFRbeta TTbeta.  Ref: 12794304 J Mol Neurosci, 2003
- METHODS Retroviral vectors were constructed to express the high affinity NGF  [ **NGFB** ]  receptor  [ **NGFR** ]  TrkA and low affinity NGF  [ **NGFB** ]  receptor  [ **NGFR** ]  p75 NTR.  Ref: 12877805 Chin Med J (Engl), 2003
- Expression of low affinity nerve growth factor  [ **NGFB** ]  receptor  [ **NGFR** ]  p75 and its regulation in spinal motor neurons of aged rats following axonal injury were investigated by immunocytochemical staining with antibody against p75.  Ref: 12876432 Dev Neurosci, 2003
- PURPOSE The purpose is to analyze the possible correlation between expression and activation of the high affinity nerve growth factor  [ **NGFB** ]  NGF  [ **NGFB** ]  receptor  [ **NGFR** ]  TrkA, cell cycle protein expression, and disease outcome in serous ovarian carcinoma.  Ref: 12796393 Clin Cancer Res< MedlineTA>Clin Cancer Res, 2003
- We present the results of cosegregation analysis for the following loci the gene for the Na, K ATPase alpha 1 subunit isoform Atp1a1, the endothelin 2 gene Edn2, the low affinity nerve growth factor  [ **NGFB** ]  receptor  [ **NGFR** ]  gene Lngfr, and a region of chromosome 10 marked with the D10Rat58 microsatellile located 3 cM away of the aldolase C gene AldC.  Ref: 12884521 Genetika, 2003
- In the present study, the effect of signaling generated from the Erb B2 homodimer as a result of transcription of the MUC1 gene was investigated in human mammary epithelial cell lines MTSV1 7 and Hb2 stably transfected with a pBAT trk neu construct in which the extracellular domain of Erb B2 was replaced with the corresponding domain from the nerve growth factor  [ **NGFB** ]  NGF  [ **NGFB** ]  receptor  [ **NGFR** ] .  Ref: 12798775 Cell Biol Int, 2003
- Purification of Schwann cells by selection of p75 low affinity nerve growth factor  [ **NGFB** ]  receptor  [ **NGFR** ]  expressing cells from adult peripheral nerve.  Ref: 12706843 J Neurosci Methods, 2003
- In both NGF  [ **NGFB** ]  transgenic and nontransgenic mice with hypomorphic expression of p75NTR  [ **NGFR** ] , there is a significant loss of DRG neurons compared to mice with normal p75NTR  [ **NGFR** ]  expression.  Ref: 12887413 Eur J Neurosci, 2003
- We could show that the viral latent membrane protein 1 LMP1 is responsible for the induction of CD83 by using an LCL expressing a ligand or antibody inducible recombinant nerve growth factor  [ **NGFB** ]  receptor  [ **NGFR** ]  LMP1 fusion protein.  Ref: 12857898 J VirolJ Virol, 2003
- This distribution is related to the spatial distribution of nerve endings identified by labeling of the low affinity nerve growth factor  [ **NGFB** ]  NGF  [ **NGFB** ]  receptor  [ **NGFR** ]  p75 NTR.  Ref: 12821716 J Dent Res, 2003
- Comparisons of calcitonin gene related peptide immunoreactivity in the dorsal horn revealed that the area occupied by DRG central processes was not significantly different between p75NTR  [ **NGFR** ]  hypomorphic mice and wild type siblings, or between NGF  [ **NGFB** ]  transgenic mice with either hypomorphic or normal expression of p75NTR  [ **NGFR** ] .  Ref: 12887413 Eur J Neurosci, 2003
- An 11 mer antisense PNA directed at the initiation codon dose dependently inhibited p75NTR  [ **NGFR** ]  expression and death signalling by nerve growth factor  [ **NGFB** ]  in Schwann cell cultures.  Ref: 12824038 Bioorg Med Chem Lett, 2003
- Here, we report a method to purify p75 low affinity nerve growth factor  [ **NGFB** ]  receptor  [ **NGFR** ]  p75LNGFr expressing Schwann cells from peripheral nerve biopsies in adult rats using magnetic activated cell separation MACS.  Ref: 12706843 J Neurosci Methods, 2003
- Quantitative in situ hybridization was used to detect changes in neuronal PACAP mRNA expression and to correlate this expression with neuronal mRNA expression of the NGF  [ **NGFB** ]  receptor  [ **NGFR** ]  tyrosine kinase trk A.  Ref: 12890505 Neuroscience, 2003
- This study examined the roles of nerve growth factor  [ **NGFB** ]  NGF  [ **NGFB** ]  and the p75 neurotrophin receptor p75NTR  [ **NGFR** ]  in the growth of dorsal root ganglion DRG central processes in the dorsal horn.  Ref: 12887413 Eur J Neurosci, 2003
- Although we demonstrate the expressions of several markers, such as CD10, CD56, CD99, factor XIII, and low affinity nerve growth factor  [ **NGFB** ]  receptor  [ **NGFR** ] , in the spindle cells of the whorls for the first time, the lineage of the whorls still cannot be addressed due to the fact that these markers are lineage nonspecific.  Ref: 12833576 Yonsei Med J, 2003
- What is important is the induction of cells that have an atypical to this pathway allure staining positive for the neurofilament 3A10 monoclonal antibody, specific for nerve cells and the anti p75 Nerve Growth Factor  [ **NGFB** ]  Receptor  [ **NGFR** ]  monoclonal antibody.  Ref: 12803109 Haematologia Budap , 2002
- Immunohistochemical staining of the explanted cords revealed that the surviving cells stained positively for nerve growth factor  [ **NGFB** ]  receptor  [ **NGFR** ] .  Ref: 12820682 J Neurotrauma, 2003
- The cells comprising whorls showed positive reactions for CD10, CD56, CD99, factor XIII, and low affinity nerve growth factor  [ **NGFB** ]  receptor  [ **NGFR** ]  in addition to vimentin and alpha smooth muscle actin.  Ref: 12833576 Yonsei Med J, 2003
